# Testing a comprehensive model of implementation and sustained use for EBTs for PTSD: A national investigation in VA residential settings

**DOI:** 10.1186/1748-5908-10-S1-A30

**Published:** 2015-08-20

**Authors:** Paula P Schnurr, Vanessa Simiola, Josef Ruzek, Richard Thompson, Rani Hoff, Joan M Cook

**Affiliations:** 1 National Center for PTSD, Department of Veterans Affairs, and Department of Psychiatry, Geisel School of Medicine at Dartmouth, White River Junction, NH, 05001, USA; 2 Department of Psychiatry, Yale School of Medicine, and National Center for PTSD, Department of Veterans Affairs, West Haven, CT, 06516, USA; 3 National Center for PTSD, Department of Veterans Affairs, and Palo Alto University, Palo Alto, CA, 94304, USA; 4 Independent Practice, Houston, TX, 77098, USA

## 

The national roll-outs of evidence-based psychotherapies in the Department of Veterans Affairs (VA) afford an unusual opportunity to study both implementation and sustainability. Although unique in some aspects of management and resources, the VA also serves as an excellent laboratory to understand the implementation of best practices as it is a more organized and controlled environment, free of the barriers faced in other more fragmented segments of the U.S. health care system. In a two-year NIMH grant, we utilized a theory-based model to collect baseline data regarding the adoption of two evidence-based treatments for Posttraumatic Stress Disorder (PTSD), Prolonged Exposure (PE) and Cognitive Processing Therapy (CPT), in a national sample of 38 VA PTSD residential treatment programs with over 200 providers. In a subsequently funded NIMH R01, we are extending our investigation with the same population and model to see: (1) how well PE and CPT are sustained over time, (2) what organizational and individual factors influence sustainability, and (3) what effects implementation and sustained use of PE and CPT have on patient outcomes. Implications for implementation in and outside of the VA health care system will be discussed.

Updates on measurement of a model of implementation for health care: advances toward a testable theory

One comprehensive theoretical model for understanding implementation of innovations was initially developed by Rogers (1962) and elaborated on by others (Greenhalgh et al. 2005). This model construed implementation as a complex process influenced by five broad constructs: (a) perceived characteristics of innovation, (b) potential adopter characteristics, (c) communication and influence, (d) system antecedents and readiness, and (e) outer context. Although a considerable evidence-base was used to develop the model, the authors did not fully operationalize their model, making it difficult to test formally. Our group undertook a systematic review of the literature and, using an iterative process, we examined existing measures and utilized or adapted items. Where no one measure was deemed appropriate, we developed other items to measure the constructs through consensus. The review and iterative process of team consensus identified three types of data that could be used to operationalize the constructs in the model: survey items, interview questions and administrative data. Over three waves of data collection concerning the implementation of two evidence-based psychotherapies disseminated nationally within Department of Veterans Affairs, we have made changes to the quantitative measurement of aspects of this model including the exclusion of the measurement of some constructs (e.g., learning style, locus of control, tolerance of ambiguity) as well as refinement of others (e.g., needs, motivation, knowledge-seeking). This presentation will review these changes as well as psychometric properties of other constructs and their items.

Testing the model using quantitative data for implementation of two evidence-based psychotherapies for PTSD in VA residential treatment programs: outcomes for two yearly time points

This study examined the implementation of two evidence-based psychotherapies, Prolonged Exposure (PE) and Cognitive Processing Therapy (CPT), in the Department of Veterans Affairs (VA) residential Posttraumatic Stress Disorder treatment programs. The current analyses focused on continued implementation a year after an initial assessment of implementation and of provider and site variables thought to be related to implementation. Seventy-five providers from 38 programs provided complete quantitative data on both baseline and follow-up. At one year follow-up, there was continued effect of supportive organizational context (i.e., dedicated time and resources and incentives and mandates) on the implementation of both PE and CPT delivered in a group format.

Unlike at baseline, effects of perceived characteristics of treatment on implementation of PE and of supportive organizational context on CPT delivered individually were no longer significant. Rather, social connections predicted a lower likelihood of implementation of CPT individually. These effects all remained, even after taking into account baseline levels of implementation.

Implementation of two evidence-based psychotherapies for PTSD in VA residential treatment programs: patient-level outcomes

This presentation will discuss the effects of implementation of two EBTs for PTSD (PE and CPT) in the U.S. Department of Veterans Affairs (VA) residential treatment programs on patients' PTSD symptom severity, alcohol and drug use, and treatment satisfaction. The instruments were administered to patients upon admission and four months post-discharge. The short form of the Mississippi Scale for Combat-Related PTSD was used to measure PTSD and alcohol and drug abuse were measured using the composite indexes from the Addiction Severity Index. Controlling for length of stay and baseline symptoms, implementation of PE and CPT predicted improvement in PTSD symptom severity and alcohol use. The implications of these findings are that two EBTs for PTSD can be feasibly and effectively disseminated to routine clinical settings and implementation produces favorable patient outcomes.

